# Predicting cervical cancer screening behavior among women in southern Iran: a cross-sectional study with PEN-3 model

**DOI:** 10.1186/s12905-023-02416-x

**Published:** 2023-05-12

**Authors:** Zahra Hosseini, Niloofar Seyrafi, Atefeh Homayuni, Ali Mouseli, Atefeh Homayuni

**Affiliations:** 1 Health Promotion and Education, Social Determinants in Health Promotion Research Center, Hormozgan Health Institute, Hormozgan University of Medical Sciences, Bandar Abbas, Iran; 2 Student Research Committee, Faculty of Health, Hormozgan University of Medical Sciences, Bandar Abbas, Iran; 3 Student Research Committee, Faculty of Health, Hormozgan University of Medical Sciences, Bandar Abbas, Iran; 4 Social Determinants in Health Promotion Research Center, Hormozgan Health Institute, Hormozgan University of Medical Sciences, Bandar Abbas, Iran

**Keywords:** Cervical cancer, Pap smear test, PEN-3 model, Screening, Women

## Abstract

**Introduction:**

Despite the fact that the Pap smear test is a simple, affordable, painless and relatively reliable method to diagnose cervical cancer in women, the majority of women are unaware of the value of this valuable diagnostic method. There are many cultural and social barriers to this diagnostic method. The present study was conducted to predict cervical cancer screening behavior with PEN-3 model among women residents of Bandar Abbas.

**Methods:**

The present descriptive-analytical study was conducted on 260 women aged 18 years and above who visited the comprehensive health centers of Bandar Abbas. The data were collected online using a demographic information questionnaire and a researcher-made questionnaire based on the PEN-3 model constructs and analyzed using Mann-Whitney U test, Pearson correlation test and logistic regression analysis in SPSS-23.

**Results:**

The participants’ age ranged between 18 and 52 years with an average of 30.95 ± 5.47 years. 27.7% of the participants had done their last pap smear test less than 1 year before the study and 26.2% had not done a pap smear test until the time of study. The results showed that the mean scores of knowledge (11.28 ± 2.87), attitude (64.96 ± 4.96), enablers (44.66 ± 5.8), and nurturers (36.02 ± 8.83) in women who had done the cervical cancer screening behavior was more than those who had not done the behavior. Also, the results of logistic regression analysis showed that knowledge, attitude and nurturers were the major predictors of cervical cancer screening behavior.

**Conclusion:**

The present findings showed that knowledge, attitude, enablers and nurturers play a major role in women’s participation in Pap smear test. These findings should be considered in the development and implementation of educational interventions.

## Introduction

Cervical cancer occurs due to an abnormal growth of cells in the cervix, which connects the uterus and vagina. It is one of the most prevalent cancers of the female reproductive system that originates from the cervix [1]. Cervical cancer is the fourth most prevalent malignancy among women worldwide [2]. The incidence of the disease increases after the age of 30 and its peak will be at the age of 65–69 [3]. In 2020, 604,000 new cases were reported worldwide and more than 341,000 women died of the disease [4]. Almost 90% of these cases have occurred in low- and middle-income countries. As predicted, if effective measures are not taken, the number of mortalities will increase by 25% in the next 10 years [5, 6]. The incidence rate of this cancer in Iran is lower than the global rate, but due to the increased consumption of cigarettes, hookah and drugs by the young generation, the change in lifestyle and the increase in risky behaviors, it is expected that HPV will increase and the incidence of this cancer will also increase in Iran [7]. According to the latest national report on the recorded cases of cancer, the incidence of cervical cancer among Iranian women has ranged between 0.36 and 3.73 per hundred thousand people [8].

The most important risk factors of this disease are having multiple sexual partners, smoking, obesity, weak immune system, oral contraceptives and failure to use condoms, early marriage, high number of births, young age at the first childbirth and social factors [9, 10].

Cervical cancer can be easily diagnosed and treated in the early stages. If the diagnosis is delayed, the possibility of its treatment is greatly reduced [11]. Cervical cancer screening (Pap smear test) is a cost-effective method that can reduce 70% of mortalities induced by this cancer in countries marked by a high uptake [12]. Despite the importance of preventive behaviors of cervical cancer, there are factors influencing the low screening and performance of Pap smear test. These factors are at four levels. One involves factors such as low literacy, being unemployed, not marrying and low income. The psycho-social factors include embarrassment, unpleasant examinations, low acculturation, mortality, language barriers, mistrust of specialists, fear of the pap smear test, fear of the results, concerns about confidentiality, lack of knowledge, discrimination and social rejection or disapproval. The external factors include not visiting a doctor, no medical insurance, high cost, restrictive work policies, payment policies, poor transportation and quality of care and screening, and cognitive-emotional processes that have a strong negative effect on extra-individual factors [13].

The culture of any society plays a leading role in reducing high-risk behaviors, and the PEN-3 model places culture at the center of beliefs, behaviors, and health outcomes [14]. The PEN-3 cultural model was developed by Dr. Collins Airhihenbuwa (1989) [15] in response to the apparent omission of culture in explaining health outcomes in the existing health behavior theories and models. Perception, enabler and nurturer model (PEN-3) is a theoretical model based on the constituent elements of the PRECEDE model, health belief and rational action of health behaviors. The “PEN-3” has three interrelated constructs, each with three factors that form the acronym PEN: (1) the first construct deals with a health education for individuals, extended family (relatives) or neighbors (communities and leaders), (2) the second construct consists of the educational diagnosis of health behavior, including perceptions (knowledge, beliefs and values), enablers (social factors affecting health behaviors) and nurturers (people who are important to the individual and he/she follows them), and (3) the third construct examines the cultural appropriateness of health behavior and includes positive, negative and intermediate beliefs [16, 17].

Bandar Abbas is the capital of Hormozgan province and one of the main ports in the south of Iran. It is located on the edge of the Persian Gulf, and hosts multicultural people from all over the country who come to earn a living. Since healthy and preventive beliefs and behaviors are formed in any society based on the social and cultural background of people in the society, it seems that exploring the social and cultural factors influencing the performance of the Pap smear test among women can provide appropriate background information to develop interventions aimed at improving screening against this cancer. Therefore, the present study aimed to predict cervical cancer screening behavior with PEN-3 model among women residents of Bandar Abbas.

## Methods & materials

### Study design and population

The present descriptive-analytical study was conducted from January 20, 2022 to April 30, 2022. The research population included all women over the age of 18 who visited the comprehensive health centers in Bandar Abbas (in the south of Iran).

### Sample size and sampling procedure

According to the previous studies and using Cochran’s formula, the sample size was estimated at 260. The inclusion criteria were: age over 18 years, having an active file in the SIB system, a history of sexual intercourse and no history of Pap smear test, or no history of regular Pap test as advised by the Ministry of Health, less than 21 years of age which at least 3 years have passed since their marriage or sexual relationship, no history of cervical cancer, willingness to participate in the study, access to the Internet to answer questions, and informed consent to participate in the study. The exclusion criterion was failure to meet any one or more of the inclusion criteria.

The data collection was done during the COVID-19 pandemic in Iran. To protect the participants’ health, the data were collected online. The hyperlink to the questionnaire was made available to the participants through the healthcare workers and health centers in the relevant social networks (Telegram and WhatsApp). Before data collection, the objectives of study were explained to the participants. The participants were assured of the anonymity of the information they provided and the confidentiality of data. They were assured that their information would be used only for research purposes.

### Instrumentation

Self-report questionnaires were used to collect the information. These questionnaires were: a demographic questionnaire, knowledge questionnaire and a researcher-made questionnaire based on the PEN-3 constructs.

The demographic information included: participant’s age, age of first sexual intercourse, marital status, number of births, education level, employment status, history of OCP use, history of venereal disease, history of extramarital relationship, and history of cancer in family and relatives.

#### Knowledge questionnaire

It included 14 questions measuring the participants’ knowledge of cervical cancer using the Pap test screening guidelines in Iran [18]. The options available as the answer to the questions were “Yes” (1 point), “No” (0 point) and “I don’t know” (0 point). The minimum and maximum scores that could be gained in this section ranged between 0 and 14, respectively.

#### PEN-3 Questionnaire

It was developed based on the constructs of the PEN3 model. This questionnaire includes 3 constructs (attitude, enablers, and nurturers). The attitude construct consisted of 16 questions (e.g., “*The Pap smear test is very effective in reducing the incidence of cervical cancer and its mortality rate*.”). The questions related to this construct were rated on a 5-point Likert scale ranging from completely agree (5 points) to completely disagree (1 point). The minimum and maximum scores that could be obtained in this section were 16 and 80, respectively. The second part of the questionnaire contained 12 questions (e.g., “*To what extent do the staff of the healthcare center and the doctor’s office have sufficient skills to examine and perform the Pap test?*”) to measure the enablers construct. The questions were rated on a 5-point Likert scale ranging from “very much” (5 points) to “very little” (1 point). The minimum and maximum scores that could be obtained in this construct were 12 and 60, respectively. The nurturers construct contained 11 questions (e.g., “*To what extent does your husband disapprove of your presence in the healthcare center and the doctor’s office to perform the Pap test?*”). The questions were rated on a 5-point Likert scale ranging from “very much” (5 points) to “very little” (1 point). The minimum and maximum scores in this construct were 11 and 55, respectively.

The only behavioral question was “*Have you ever had a Pap test?*”. Answers were provided as “Yes” (1 point) or “No” (0 points). The answer “Yes” indicated performing the behavior and the answer “No” meant not performing the behavior.

In all three mentioned constructs (attitude, enablers and nurturers), a higher score means a better and more positive attitude, more enablers (e.g., access to medical centers, health insurance coverage, etc.), and more nurturers (e.g., health workers and doctors, mass media, etc.).

### Data analysis

In this study, SPSS 23 was used for data analysis. Descriptive statistics were used such as mean and standard deviation. Smirnov-Kolmogorov’s test was used to check the normality of data. As for inferential statistics, Mann-Whitney U test, Pearson’s correlation test and logistic regression analysis were used.

### Ethical considerations

The present study was approved by the ethics committee of Hormozgan University of Medical Sciences (#IR.HUMS.REC.1399.007). The research participants were informed that participation in this study was completely voluntary and they could withdraw from the study any time they wanted. They were also assured that all the collected data would remain confidential. A written informed consent was obtained from all participants.

## Results

### Demographic characteristics

A total number of 260 participants completed the questionnaires. Their age ranged between 18 and 52 years with an average of 30.95 ± 5.47 years. The participants’ first experience of sexual intercourse was between 12 and 33 years of age with an average of 20.22 ± 2.83 years. The participants’ other demographic information is summarized in Table 1.

**Table 1:** Research participants’ demographic characteristics

Characteristics	Categories	n(%)
Marital status	Single	5 (1.9)
	Married	248 (95.4)
	Widow	6 (2.3)
	Divorced	1 (0.4)
Level of education	Under diploma	29 (11.1)
	Diploma	147 (56.5)
	Associate degree	27 (10.4)
	Bachelor’s degree	51 (19.6)
	Master’s degree and higher	6 (2.3)
Job situation	University student	7 (2.7)
	Retired	2 (0.8)
	Housewife	224 (86.2)
	Employee	27 (10.4)
Deliveries	No children	15 (5.8)
	1	91 (35)
	2	114 (43.8)
	3	29 (11.2)
	4	9 (3.5)
	5	2 (0.8)
Taking birth control pills	Yes	154 (59.2)
	No	106 (40.8)
History of extramarital relationship	Yes	5 (1.9)
	No	255 (98.1)
History of venereal disease	Yes	201 (77.3)
	No	59 (22.7)
History of cancer in family and relatives	No family history	255 (98.1)
	2nd degree relatives	4 (1.5)
	degree relatives 3rd	1 (0.4)
Last Pap Smear	Less than 1 year ago	72 (27.7)
	3 years ago	14 (5.4)
	3–5 years ago	68 (26.2)
	More than 5 years ago	38 (14.6)
	I have not done it yet	68 (26.2)
Behavior	Yes	192 (73.8)
	No	68 (26.2)

Table 2 showed the comparison between research variables (knowledge, attitude, enablers and nurturers) according to the behavior variable. The distribution of the research variables using Kolmogorov-Smirnov’s test indicated that the data were not normal and the significance level of all the research variables was less than 0.05. Therefore, the Mann-Whitney U test was used to make a comparison. The results indicated in Table 2 show that the significance level of the above variables is less than 0.05 and the mean scores of knowledge, attitude, enablers and nurturers varied significantly according to the behavior variable. Moreover, in all cases, for these variables, the mean scores of those who answered “Yes” to the behavior question were significantly higher than the mean scores of those who answered “No” to the behavior question.

**Table 2:** U-Man-Whitney U test of the research variables according to the behavior variable

	Behavior	Number	Mean	Standard Deviation	Mann-Whitney U	p. value
Knowledge	No	68	7.2353	2.56915	2187.500	0.000
	Yes	192	11.2812	2.87871		
Attitude	No	68	60.3971	4.04135	2732.500	0.000
	Yes	192	64.9583	4.96146		
Enablers	No	68	39.1176	4.89145	3212.500	0.000
	Yes	192	44.6667	5.80064		
Nurturers	No	68	27.4853	3.26671	2766.500	0.000
	Yes	192	36.0260	8.83083		

Table 3 showed the correlation test results of the research variables. The results showed that knowledge, attitude, enablers and nurturers have a positive linear correlation with each other.

**Table 3:** Correlation coefficients of research variables

	Knowledge	Attitude	Enablers	Nurturers
**Knowledge**	1			
**Attitude**	0.482^**^	1		
**Enablers**	0.596^**^	0.539^**^	1	
**Nurturers**	0.678^**^	0.522^**^	0.713^**^	1

Tables 4 and 5 represented the results of the logistic regression test to test the hypothesis that the research variables (knowledge, attitude, enablers and nurturers) affect individuals’ behavior. The reason for using logistic regression is that the behavior variable is dichotomous.

Table 4 represented the prediction model if all the independent variables (knowledge, attitude, enablers and nurturers) were included in the model and contends that although in reality 68 people answered “No” (i.e., not showing the behavior), 37 individuals were predicted to answer “Yes” to the behavior. Therefore, 54.4% of the prediction model was correct (model identification) and out of 192 people who answered “Yes”, 172 individuals were predicted to have answered “Yes” to the behavior, which is 89.6% of the correct prediction (sensitivity). In total, the accuracy of the model is 80.4%.

**Table 4:** Prediction classification with the mere inclusion of research variables (knowledge, attitude, enablers and nurturers)

Block 1		Predicted		
		Behavior		
		No	Yes	Coverage percentage
Behavior	No	37	31	54.4
	Yes	20	172	89.6
Coverage percentage				80.4

Table 5 presented the results of testing the presence of research variables. In block 1, the significance level of the variables of knowledge, attitude and nurturers is less than 0.05. Therefore, these three variables are present in the model and the enablers should be removed from the model. The odds ratio of predicting the variables of knowledge, attitude and nurturers is greater than 1. Therefore, these variables are influential.

**Table 5:** Model variables in block 1 (presence of all research variables (i.e., knowledge, attitude, enablers and nurturers)

B		B	S.E	Wald	df	Sig	Exp (B)
Block 1	Knowledge	0.342	0.082	17.395	1	0.000	1.407
	Attitude	0.190	0.070	7.445	1	0.006	1.209
	Enablers	0.043	0.045	0.911	1	0.340	1.044
	Nurturers	0.124	0.052	5.757	1	0.016	1.132

## Discussion

The present study aimed to predict cervical cancer screening behavior with PEN-3 model among women residents of Bandar Abbas. The results showed that the mean score of knowledge in women who performed cervical cancer screening behavior was higher than those who did not show the healthy behavior. This finding is consistent with a body of research conducted by Anaman-Torgbor et al. [19], Adunlin et al. [20], Kwok et al. [21], Hislop et al. [22], Juon et al. [23], Kirubarajan et al. [24], Momeni et al. [25], Bakht et al. [26], Enjezab et al. [27], Keshavarz et al. [28], and Akbari et al. [29]. In their study, Wong et al. [30] mentioned the most common reason for not taking the test to be inadequate knowledge of the necessity of taking the test and its benefits. In another study by Enjezab et al. [27], among the most common causes of women avoiding diagnostic tests for common female cancers, the following were raised: lacking knowledge of the ever existence of these tests, lacking knowledge of the healthcare centers that offer these tests and train on them, lacking knowledge of the free tests offered by the health staff, lacking knowledge of the preventability of cancer, lacking knowledge of the test method and lacking training of health staff to appropriately perform these test, and one of the most motivating reasons for performing screening tests was increasing awareness through mass media. The results of Adunlin et al.‘s [20] study on immigrants to the United States showed that awareness of the disease and access to information sources were the facilitators of screening. Kwok et al. [21] in a study on Australian-Chinese women showed that women’s awareness of cervical cancer was low and few participants perceived the benefits and purpose of screening. The results of another study by Vega Crespo et al. [31] showed that increasing awareness of cervical cancer screening in communities and families increases the acceptance of the Pap test and reduces the negative social labeling. Including husbands in preventive programs can improve screening by removing the barriers caused by the lacking knowledge of screening and pessimism in those communities.

The results showed that the mean attitude scores of women who performed the cervical cancer screening behavior were higher than those who did not show this behavior. This finding is consistent with the findings of studies conducted by Hislop et al. [22], Vega Crespo et al. [31], Momeni et al. [25], Moradi et al. [32], Fallahi et al. [33], Shakibazadeh et al. [34] and Kirubarajan et al. [24]. In a study conducted by Vega Crespo et al. [31] among women undergoing screening in Ecuador, the following were found to be the cervical cancer screening barriers: fear of pain during the test, embarrassment (an uncomfortable and even humiliating experience, especially when a male doctor is present), fear of exposure to misbehavior or sexual harassment during the test, fear of rape, negative social labeling for doing the Pap test (because some women think the screening is mainly for women who have multiple sexual partners and it is not considered necessary for respectable women, and participation in regular screening can lead to social discrimination), fear of the possible positive result and preference for not doing the screening, a fear induced by the lacking knowledge. The results of another study conducted by Kirubarajan et al. [24] showed that the fear of pain/discomfort during the Pap test, feeling embarrassed, fear of the side effects of screening and fear of possible cancer diagnosis were some barriers to screening. In Hislop et al.‘s [22] study on Chinese-Canadian women, the belief that the Pap test can help prevent cancer and that the Pap test is necessary for asymptomatic women were associated with a regular performance of the test. Among the beliefs that form the non-testing of women participating in the study of Fallahi et al. [33] were the belief that single women do not get the cancer, the false belief that cervical cancer cannot be prevented, and the false belief about the chances of transmitting infection through the test. As perceived by the present participants, the importance of health and having a positive attitude about it were effective in doing the test.

The research findings showed that the mean enablers score in women who showed the cervical cancer screening behavior was higher than those who did not show this behavior. This finding is consistent with a body of research by Adunlin et al.[20], Anaman-Torgbor et al. [19], Kwok et al. [21], Hislop et al. [22], Kirubarajan et al. [24], Bahmani et al. [35], Momeni et al. [25], Fallahi et al. [33], Shakibazadeh et al. [34], Vega Crespo et al. [31]. In Kirubarajan et al.‘s study [24], low access to services, difficulty finding a consistent healthcare provider, especially after moving away from home for work or school, especially in rural areas with only one provider, or places with limited access to women doctors, the transportation system, the cost of screening services, and financial and time limitations were some barriers to screening. Another study by Anaman-Torgbor [19] on African immigrant women living in Brizin, Australia revealed some other barriers such as the absence of warning signs, concerns about the service provider’s gender, lack of privacy, cultural and religious beliefs (the fact that this test is done on the vaginal area which is an intimate part of body not to be seen or touched by anyone other than the sexual partner. The women participants believed that such a level of intimacy should only be shared with husbands or sexual partners). The healthcare system related factors were also found to be the barriers to the screening behavior. Vega Crespo et al. [31]. showed that the long waiting time, difficulty accessing health centers, distrust in health centers and shortage of time for screening due to family obligations, social inequality (the poor and uneducated women’s less access to screening) were some barriers to screening. The results of a qualitative study by Bahmani et al. [35] indicated that wrong cultural beliefs (such as being strong and healthy, having a clean womb) and wrong religious beliefs (such as the destiny of humans being in the hands of God and cancer being a worldly punishment) are among the factors affecting the rate of healthy behavior. Among the needs and demands the participants mentioned in Fallahi et al.’s [33] study were increasing the appropriate and efficient interaction of the health staff in clinics and the healthcare centers. Using female staff in health-care centers, making the screening test mandatory and giving a day off to working women to perform the test, were among the motivating factors for the screening test. Also, the lack of economic well-being and the ability to pay the cost of the test, the lack of insurance coverage for screening behaviors, and the lack of compensation for testing costs were among the issues the participants mentioned to be solved to facilitate the Pap test. The other cultural barriers to the performance of the Pap test were women’s embarrassment in the presence of a male physician and men’s opposition to the test due to religious beliefs.

The results showed that the mean score of nurturers in women who showed the cervical cancer screening behavior was higher than those who did not show the behavior. This finding is consistent with a body of research by Adunlin et al. [20], Enjezab et al. [27], Kwok et al. [21], Juon et al. [23], Hislop et al. [22], Fallahi et al. [33], Shakibazadeh et al. [34] and Vega Crespo et al. [31]. The results of Adunlin et al.‘s [20] study showed that poor access to services, lack of interpreter services, and insensitivity to patient needs were among the systemic barriers to screening. On the other hand, access to information sources and doctor’s advice and social networks were among the facilitators of screening. In this study, health insurance coverage was an important predictor of the preventive behavior of screening. Kwok et al. [21] found that a doctor’s advice was a strong motivation to perform cervical cancer screening among Chinese-Australian women. Receiving a reminder message and not paying the fee encouraged the participants to participate in the screening. In a study conducted on Korean-American women, Juon et al. [23] showed that a doctor’s advice, having health insurance, and having friends or family members who have had the Pap test are important facilitators of going regularly for the Pap test. The participants in a study by Fallahi et al. [33] pointed out the need for support and cooperation of family members, especially the husband, in performing the test. Men need to pay more attention to women’s health, motivate and encourage their wives to perform the screening tests, especially the Pap test. Husbands’ inadequate attention to women’s health, lack of financial support for testing, and lack of information and support from other family members for the test were among the main issues that the participants raised in this study. In another study conducted by Vega Crespo et al. [31], the political and religious leaders’ support in society was mentioned as a facilitator of cervical cancer screening.

The limitations of the present study include the participants’ self-reports, online data collection, and not investigating women who did not go to the healthcare centers. One strength of the study is that it was pioneering in adopting the PEN-3 model to prevent cervical cancer in Iran and Bandar Abbas.

## Conclusion

In order to improve women’s health and make an early diagnosis of cervical cancer, it is essential to know and explore the determinants of screening behavior. As the present findings showed, knowledge (knowledge of the preventability of cancer, knowledge of the need to perform the Pap test and its benefits, etc.), attitude (fear of the potential cancer diagnosis, belief in the role of destiny and fate in affliction with cancer, sense of embarrassment, etc.), enablers (cost of screening services, financial limitations, time limitations, wrong cultural and religious beliefs, health workers inappropriate behavior, etc.) and nurturers (support and cooperation of family members especially the husband, role of mass media, doctor’s advice, support and encouragement of religious and social leaders, etc.) have been effective in embracing the Pap test screening program in this study. As a result, policy makers and those deciding for the health system especially women’s health should pay enough attention to the role of these factors in the development of interventions. They should also raise the audience’s knowledge of the importance of screening and pave the way for an early diagnosis of cancer, and motivate women to perform a regular screening.

## Data Availability

The datasets generated and analyzed during the current study are not publicly available due to confidentiality and privacy related issues but are available from the corresponding author on reasonable request.
